# New Associations with the HIV Predisposing and Protective Alleles of the Human Leukocyte Antigen System in a Peruvian Population

**DOI:** 10.3390/v16111708

**Published:** 2024-10-30

**Authors:** Daisy Obispo, Oscar Acosta, Maria L. Guevara, Susan Echavarría, Susan Espetia, María Dedios, Carlos Augusto Yabar, Ricardo Fujita

**Affiliations:** 1 Centro de Genética y Biología Molecular, Facultad de Medicina Humana, Universidad de San Martín de Porres, Lima 15001, Peru; dobispoa@usmp.pe (D.O.);; 2 Facultad de Farmacia y Bioquímica, Universidad Nacional Mayor de San Marcos, Lima 15001, Peru; 3 Laboratorio de Referencia Nacional de Virus de Transmisión Sexual–Instituto Nacional de Salud, Facultad de Medicina Humana, Universidad de San Martín de Porres, Lima 15001, Peru; 4 Laboratorio de Virología Clínica y Molecular, Facultad de Ciencias Biológicas, UNMSM, Lima 15001, Peru; 5 Hospital Santa Rosa, Lima 15001, Peru

**Keywords:** HIV, HLA genes, WES, susceptibility, Peruvian population

## Abstract

The accurate determination of an individual’s unique human leukocyte antigen (HLA) allele holds important significance in evaluating the risk associated with autoimmune and infectious diseases, such as human immunodeficiency virus (HIV) infection. Several allelic variants within the HLA system have been linked to either increased protection or susceptibility in the context of infectious and autoimmune diseases. This study aimed to determine the frequency and association of HLA alleles between people living with HIV (PLHIV) as the case group and Peruvian individuals without HIV with high-risk behaviors of sexually transmitted diseases as the control group. Whole exome sequencing (WES) was used to determine high-resolution HLA allelotypes using the OptiType and arcas HLA tools. The HLA alleles present in HLA classes I (A, B, and C loci) and II (DPB1, DQA1, DQB1, and DRB1 loci) were determined in a cohort of 59 PLHIV (cases) and 44 individuals without HIV (controls). The most frequent HLA alleles were A*02:01, DPB1*04:02, and DQB1*03:419 at 36%, 30%, and 28% prevalence in general population. We found that C*07:01 (*p* = 0.0101; OR = 10.222, 95% IC: 1.40–74.55), DQA1*03:02 (*p* = 0.0051; OR = 5.297, 95% IC: 1.48–19.02), and DRB1*09:01 (*p* = 0.0119; OR = 4.788, 95% IC: 1.39–16.44) showed an association with susceptibility to HIV infection, while DQB1*03:419 (*p* = 0.0478; OR = 0.327, 95% IC: 0.11–0.96) was associated with protection from HIV infection. Our findings contribute to the knowledge of HLA allele diversity in the Peruvian population (around 70% South American indigenous ancestry) lays the groundwork for further valuable large-scale use of HLA typing and offers a novel association with HIV infection that is relevant to vaccine studies.

## 1. Introduction

According to the estimation of the Joint United Nations Programme on HIV/AIDS (UNAIDS), there are 39 million people living with HIV (PLHIV), and 29.58 million of them have access to antiretroviral therapy (ART) around the world. Of these, 2.2 million are from Latin America [1], emphasizing the substantial public health burden of HIV in this region and underscoring the critical need to understand the factors that influence HIV transmission and disease progression.

The human leukocyte antigen (HLA) is situated in a region spanning approximately 4 million base pairs on the short arm of human chromosome 6 (6p21.3). This region encompasses over 200 protein-coding genes, [2] with more than 35,000 alleles present in the HLA locus [3,4,5]. HLA class I and class II genes play a central role in host adaptive immune responses to infectious pathogens. HLA class I genes are also involved in the host innate immune response. Class I genes encode proteins that facilitate the presentation of antigenic peptides from pathogens to CD8+ T cells, leading to the destruction of infected cells and pathogens, while HLA class II antigens contribute to the initiation of adaptive immune responses by presenting antigenic epitopes to CD4+ T cells. The remarkable polymorphism of HLA class I and class II genes contributes to the diverse host immune responses to many infectious pathogens [6].

In recent years, advancements in next-generation sequencing (NGS) have brought significant improvements, allowing for the rapid, high-throughput sequencing of genomes, exomes, and targeted gene panels, with the ability to process multiple samples simultaneously. Within this framework, whole exome sequencing (WES) can be adapted for various configurations, tailored to clinical or research needs, particularly for HLA alleles determination [7]. The incorporation of NGS, a sophisticated, high-resolution assay, has been a significant advancement in HLA typing and has markedly improved the performance and data collection of many HLA laboratories [3,8,9].

The role of HLA alleles in HIV infection has been widely studied, revealing that certain alleles are associated with slower progression to AIDS, while others may increase susceptibility to HIV acquisition or accelerate disease progression. Understanding these genetic factors is essential for developing tailored HIV treatments and vaccines that consider genetic diversity across populations. However, data on HLA diversity and its impact on HIV infection remain limited in Latin American populations, particularly in Peru, where unique genetic backgrounds and distinct HIV epidemiology are present. Studying this underrepresented population is crucial, as these findings will contribute valuable insights into region-specific immune responses to HIV.

Our study aims to characterize the HLA allele distribution in a cohort of Peruvian HIV-positive individuals and to explore how these alleles might influence immune responses to HIV. By focusing on this underrepresented population, our findings will contribute to a more comprehensive understanding of HLA-mediated HIV immune responses, potentially informing the development of targeted therapeutic and preventive strategies suited to the specific needs of the Latin American population.

## 2. Materials and Methods

### 2.1. Study Population and Design

We conducted a cross-sectional study involving 59 PLHIV from the Santa Rosa Hospital (Lima, Perú) and 46 individuals without HIV with high-risk behaviors from the NGO “MCC Voluntades Lima Norte” (Lima, Perú). The study participants were enrolled from December 2019 to October 2021.

The eligibility criteria included being of Peruvian nationality, aged 18 years or older, male or female, and giving written informed consent. HIV-seropositive individuals were referred to medical care and assigned to the PLHIV group. The inclusion criteria for the no-HIV group were a negative rapid HIV test, exhibiting high-risk sexual behaviors, and perceptions of HIV risk. Transgender women who met the eligibility criteria were included in this group.

Data from patients (socio-demographic, clinical, immunological, and laboratory results) were obtained from the clinical history of each patient, and the CD4 T cell count results were provided by the National Reference Laboratory for STD/HIV-AIDS at the Instituto Nacional de Salud (INS) in Lima.

For the no-HIV group, all individuals completed a brief questionnaire about their high-risk sexual behaviors regarding HIV transmission, such as their history of sexual contact with sex workers, diagnosis or treatment of a sexually transmitted infection (STI), condom use, and having multiple sexual partners.

### 2.2. Biological Samples and DNA Extraction

Blood samples were collected in 3 mL ethylenediaminetetraacetic acid (EDTA) tubes from all the enrolled participants in both groups, and DNA was extracted using the salting-out procedure with minor modifications [10]. After digestion with Proteinase K, nucleic acid precipitation was performed initially using 7.5 M ammonium acetate and absolute ethanol, followed by a secondary precipitation with sodium chloride. This two-step approach was applied to enhance DNA purity and yield. The samples were then quantified and checked for purity using a NanoDrop spectrophotometer (Thermo Scientific, Waltham, MA, USA).

### 2.3. Whole-Exome Sequencing (WES)

Extracted DNA was sent to two different service providers, who carried out library construction and WES: Macrogen (Seoul, Republic of Korea) and Novogene (Cambridge, UK). DNA libraries were prepared using the SureSelect XT Human All Exon V6–bait library: S07604514 (Agilent Technologies, Santa Clara, CA, USA), and WES was performed using 150 bp paired-end reads and sequenced on the Illumina NovaSeq 6000 platform (Illumina, San Diego, CA, USA) according to the manufacturer’s instructions.

### 2.4. Bioinformatics Pipeline to HLA Typing

We received the exome data from both providers, generated under identical experimental conditions, and processed them through our in-house bioinformatics pipeline for HLA typing. First, the WES FastQ files were preprocessed by fastp (version 0.23.2) [11] and then mapped to the human reference genome GRCh37/hg19 using BWA software (version 0.7.17-r1188, bwa mem) [12]. The filtered and mapped reads were sorted by coordinates using SAMtools (version 1.7), [13] and possible PCR duplicate reads were marked by Picard (version 2.27.4, Broad Institute). The reads that matched the targeted chromosome 6 sequences were extracted from the bam file and mapped on a comprehensive reference panel from the IMGT/HLA database (v3.52.0) [4]. In addition, we evaluated the germline variants (single-nucleotide polymorphisms [SNPs] and small insertions/deletions [indels]) in the HLA region.

We performed the HLA typing analysis with the reads from chromosome 6 that were generated as an input. We imputed HLA class I alleles for three loci (HLA-A, HLA-B, and HLA-C) using OptiType software (version 1.3.3) [14], and HLA class II alleles for four loci (HLA-DPB1, HLA-DQA1, HLA-DQB1 and HLA-DRB1) were imputed with arcasHLA [15]. The HLA class I and class II alleles were assigned at the 2-field resolution. We set a threshold for the depth (≥20×) of HLA alleles, and samples with a depth lower than 20 were excluded. After these considerations, we included 56 cases and 44 controls.

### 2.5. Statistical Analysis

Socio-demographic and clinical data were expressed as means ± standard deviations. Comparisons between PLHIV and no-HIV groups were performed using the chi-squared test for categorical/qualitative variables and the Mann–Whitney U test (or Student’s *t*-test) for quantitative variables. Likewise, HLA allele frequencies and HLA association analysis was performed for case–control studies using additive logistic regression models. Age, sex, and CD4 values were included as covariates, and linkage disequilibrium (LD), pairwise, were determined with the PyHLA package [16].

In all analyses, a *p*-value of less than 0.05 and a confidence interval of 95% were considered statistically significant. R software (version: 4.3.1) was used for statistical analysis and plot creation.

### 2.6. Hardware and Software Environment

All software was run according to instructions on the Linux server (Ubuntu 18.04) with the following hardware configuration: Intel(R) Xeon(R) CPU E5-2697 v2 @ 3.50 GHz, each with 24 physical CPU cores, and 94 GiB RAM installed. All computational resources were provided by the Research Center of Genetic and Molecular Biology–Universidad de San Martin de Porres.

### 2.7. Ethics Statement

This study received ethical approval from the “Comité de Ética en Investigación de la Universidad de San Martín de Porres” (IRB IORB00003251 OHRP/FDA). Likewise, this study was also approved by the Ethics Committees of the following institutes that participated in the study: the National Institute of Health of Peru and Santa Rosa Hospital. Written informed consent from the patients was obtained locally at Santa Rosa Hospital and the NGO “MCC Voluntades Lima Norte”. An explanation session for each participant was held to clarify doubts about the procedure and to explain the results obtained.

## 3. Results

The study sample included 59 PLHIV (cases) and 46 no-HIV (control) subjects. The relevant socio-demographic, clinical, and ART treatment characteristics are provided in Table 1; the distribution of these characteristics varied according to gender, age, and CD4 cell count between the case and control groups. The majority of participants were male (73.0%) and born in Lima (71.43%). The median age was 41 years in the case group and 36 years in the control group. At the time of enrollment, the median CD4 count was 634 cells/mm^3^ and 952.67 cells/mm^3^ in the PLHIV and no-HIV groups, respectively.

Based on the inclusion criteria concerning HIV risk behaviors presented in the no-HIV group, all participants reported having sex with sex workers. Of these participants, 26 (56.52%) self-identified as heterosexual, 14 (30.43%) as homosexual, and 6 (13.04%) as bisexual. Regarding sexual behavior, 76% of participants reported having had multiple sexual partnerships (5–15 sexual partners in the last 12 months).

High-resolution HLA typing was performed in all 105 enrolled subjects to characterize2-field resolution HLA alleles for HLA-A, HLA-B, and HLA-C in class I, and HLA-DPB1, HLA-DQA1, HLA-DQB1, and HLA-DRB1 in class II. The number of HLA alleles present across the seven HLA loci is provided in Table 2. A total of 1394 2-field HLA alleles were observed across the seven loci in the population study. In HLA alleles class I, 200, 200, and 198 alleles were counted in HLA-A, HLA-B, and HLA-C genes, respectively (Table 2). In class II alleles, 200, 196, 200, and 200 alleles were counted in HLA-DPB1, HLA-DQA1, HLA-DQB1, and HLA-DRB1 genes, respectively (Table 2).

The three most frequent alleles for each HLA locus were A*02:01 (35.5%), A*24:02 (11.0%), and A*02:11 (10.0%); B*35:01 (11.5%), B*48:01 (6.50%), and B*15:04 (6.0%); C*04:01 (22.73%), C*07:02 (18.69%), and C*01:02 (12.12%); and DPB1*04:02 (29.50%), DPB1*04:01 (16.00%), DPB1*14:01 (13.50%), DQA1*03:01 (22.45%), DQA1*05:03 (11.73%), DQA1*04:01 (11.22%), DQB1*03:419 (28.00%), DQB1*03:02 (21.00%), DQB1*03:96 (10.00%), DRB1*09:01 (14.50%), DRB1*04:07 (13.00%), and DRB1*08:02 (11.0%) prevalence in the general population (complete HLA allele frequencies are detailed in Supplementary Table S1).

We identified three novel SNP-type variants in two patients and one control, respectively: NM_002116.8(HLA-A):c.619 + 8G > T in splice region variant (Qual:16.5), NM_001243961.2(HLA-DBQ1):c.109 + 71G > C in region variant (Qual:11.7), and NM_002117.6(HLA-C):c.620-177G > T in region variant (Qual:10). Additionally, we detected one novel deletion variant in one patient, NM_002117.6(HLA-C):c.1097-21del, in the intron variant. However, the novel variants had a variant quality score below 20. No novel alleles were identified in the evaluated HLA genes; all detected alleles had already been reported in the IPD-IMGT/HLA database.

**Figure 1:**
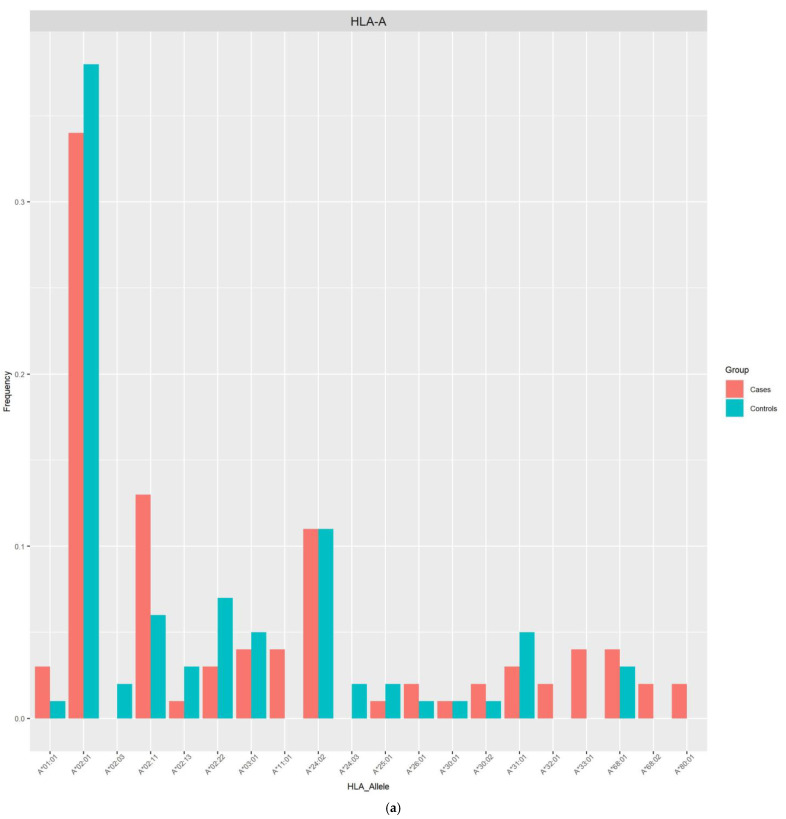

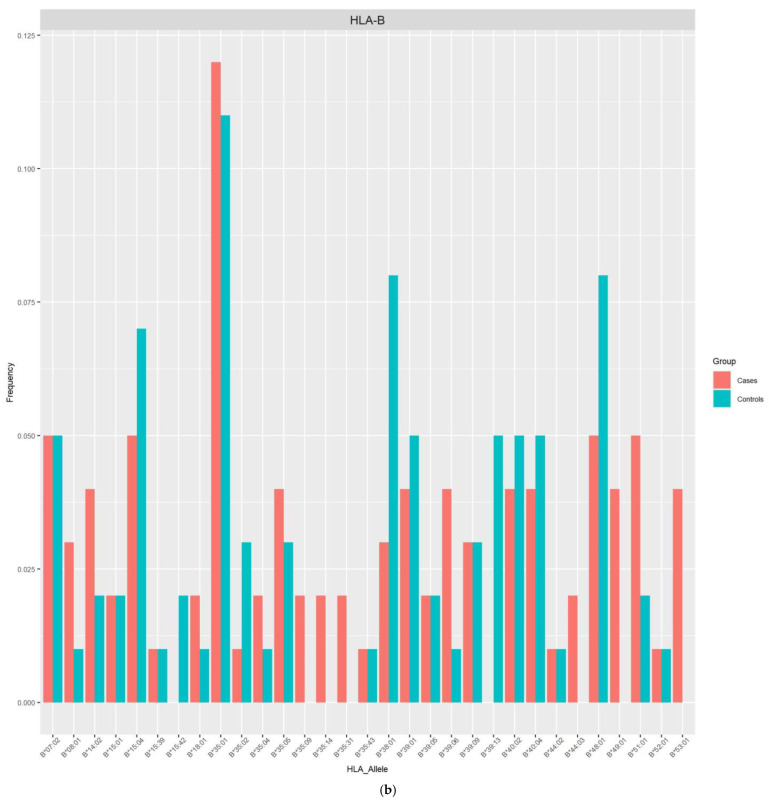

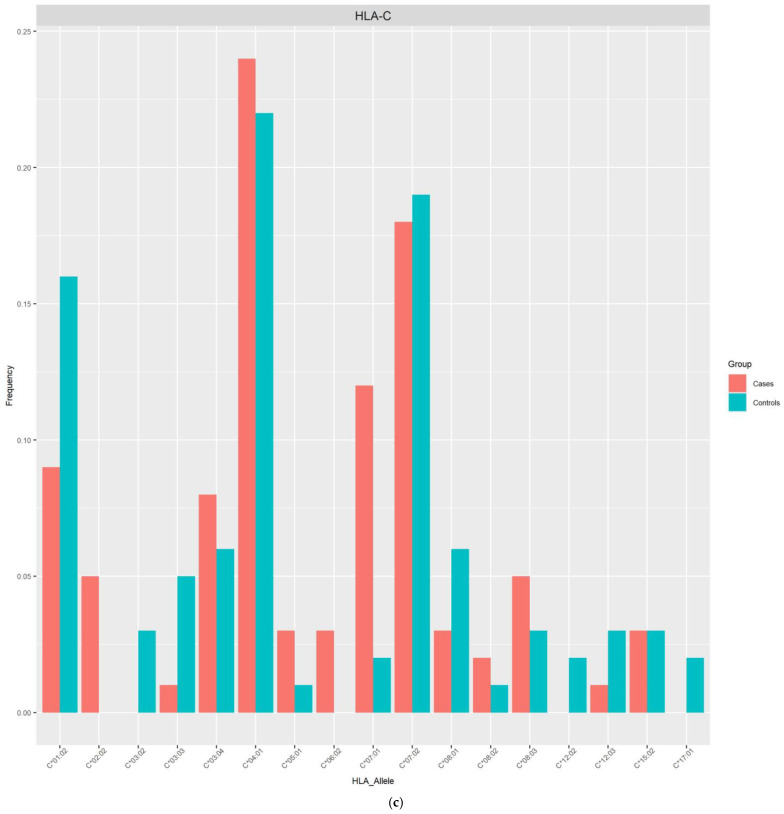
Frequency distribution of HLA class I alleles in PLHIV and those without HIV based on 2-field resolution HLA imputation. (**a**) Allele frequencies across 20 HLA-A loci. (**b**) Allele frequencies across 31 HLA-B loci. (**c**) Allele frequencies across 17 HLA-C loci.

**Figure 2:**
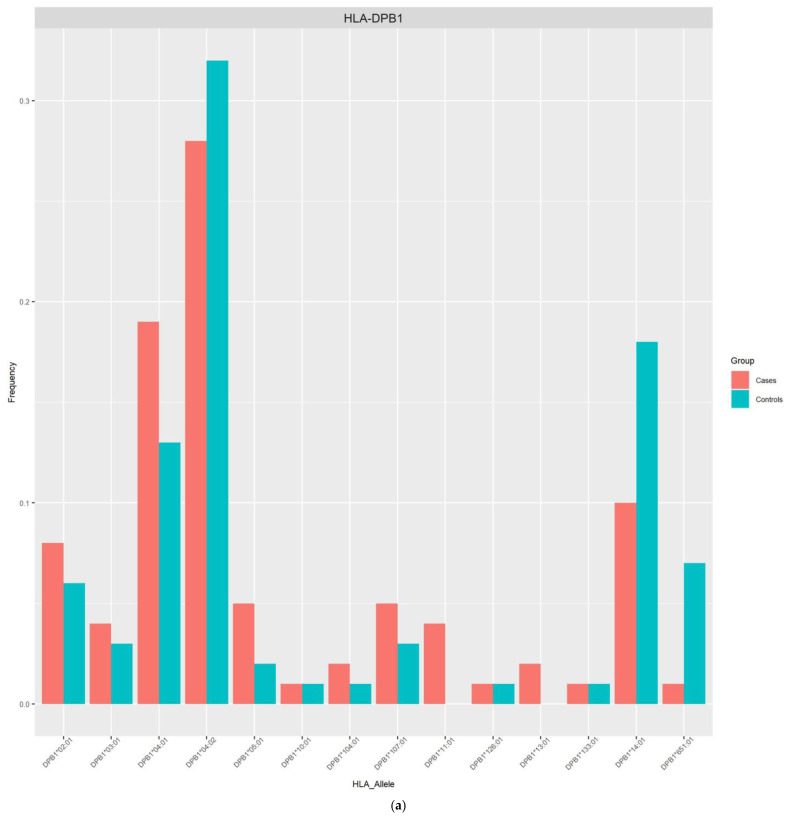

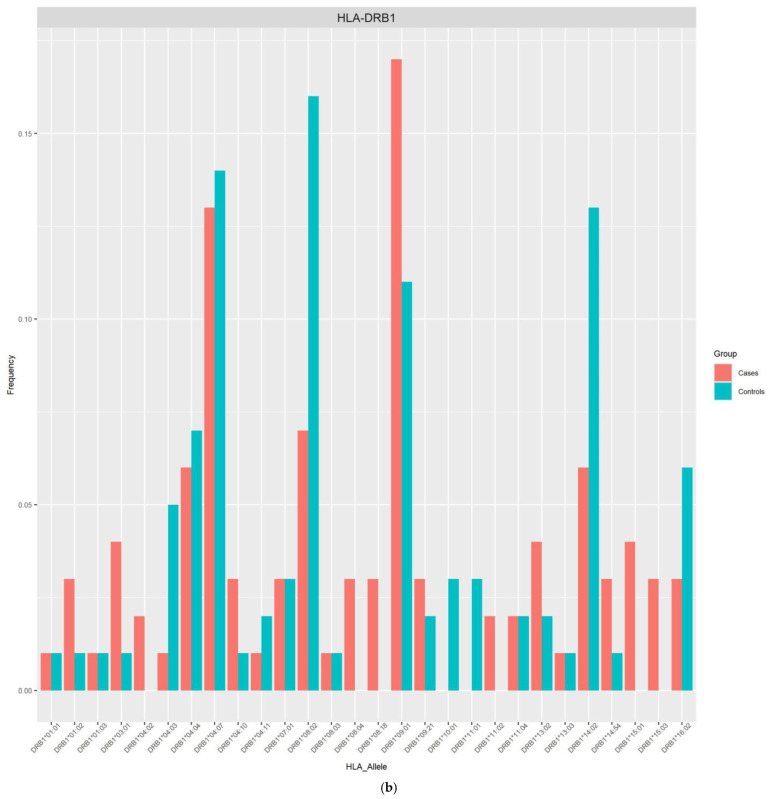

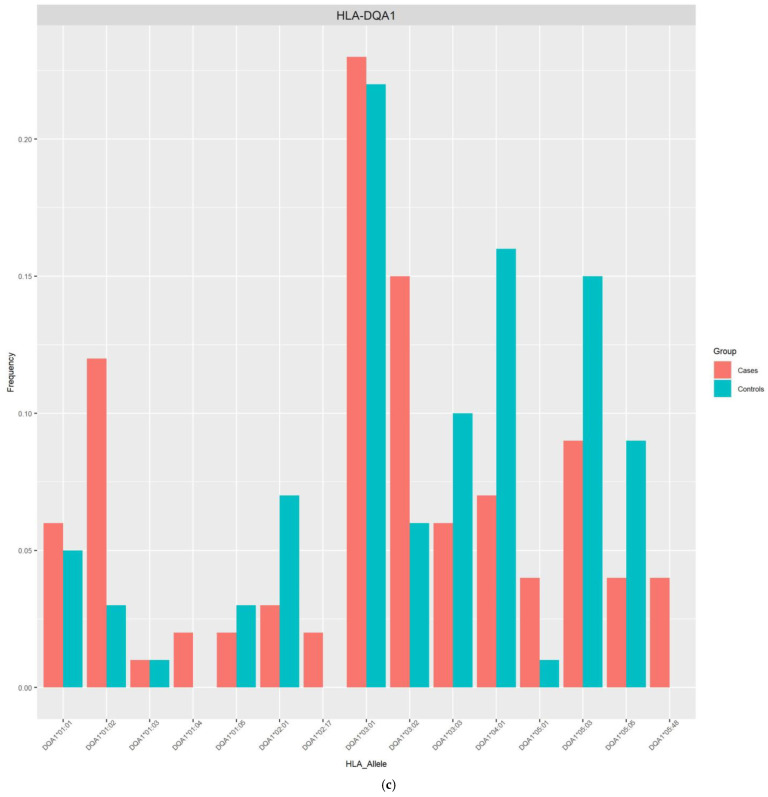

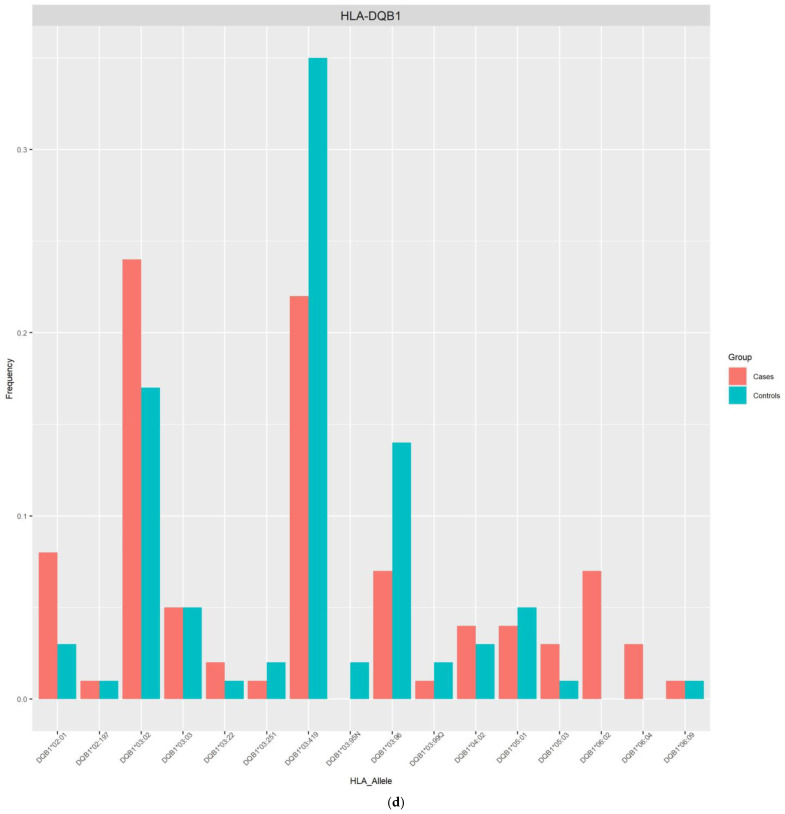
Frequency distribution of HLA alleles class II in PLHIV and those without HIV based on 2-field resolution HLA imputation. (**a**) Allele frequencies across 14 HLA-DPB1 loci. (**b**) Allele frequencies across 28 HLA-DRB1 loci. (**c**) Allele frequencies across 15 HLA-DQA1 loci. (**d**) Allele frequencies across 16 HLA-DQB1 loci.

The allele frequencies of class I (Figure 1) and class II (Figure 2) in the PLHIV sample (pink bar) were compared to those in the samples without HIV (sky-blue bar). Alleles with frequencies less than 1% were omitted.

We found four HLA alleles that were associated with HIV infection; these are shown in Table 3 (results for other HLA alleles are detailed in Supplementary Table S2). The HLA-C, HLA-DQA1, and HLA-DRB1 alleles associated with the risk of HIV infection were C*07:01 (*p* = 0.0101, OR = 10.222), DQA1*03:02 (*p* = 0.0051, OR = 5.297), and DRB1*09:01 (*p* = 0.0119, OR = 4.788), respectively; the HLA-DQB1*03:419 (*p* = 0.0412, OR = 0.3273) allele can be considered a protective factor in this sample (Table 3).

The LD for the HLA allele pairs C*07:01-DPB1*651:01 (*p* = 0.2143), C*07:01-DQA1*01:02 (*p* = 0.4433), and DPB1*651:01-DQA1* 01:02 (*p* = 1.000) do not indicate significance.

## 4. Discussion

In this study, we performed high-resolution HLA allele calling using a WES approach in a sample of a Peruvian population. HLA typing through WES proves to be a powerful tool that offers a comprehensive view of an individual’s genetic composition. This approach allows for the identification of variants in HLA genes and other genes associated with susceptibility to HIV infection. Additionally, it is considered a cost-effective strategy for genomic sequencing projects [17].

Our results show diverse HLA profiles and frequencies between PLHIV and those without HIV. We analyzed the association of HLA class I and II alleles to determine the relationship of HLA alleles that confer HIV protection or susceptibility in our population. We detected HLA alleles for three class I loci (HLA-A, HLA-B, HLA-C) and four loci in class II (HLA-DPB1, HLA-DQA1, HLA-DQB1, and HLA-DRB1) because these genes have crucial roles in antigen presentation and high degrees of polymorphism [18]. HLA allele composition is key for association studies and helps us understand the genetic risk of autoimmune and infectious diseases, such as HIV infection [6].

The relevant socio-demographic, clinical, and ART treatment characteristics varied in distribution according to gender, age, and CD4 cell count between the case and control groups. The majority of participants were male (73.0%) and born in Lima (71.43%). The median age was 41 years in the case group and 36 years in the control group. At the time of enrolment, the median CD4 count was 634 cells/mm^3^ and 952.67 cells/mm^3^ in the PLHIV and no-HIV groups, respectively. This finding is consistent with a study carried out in Peru, where 162 patients with HIV were recruited, the median age was 42 years, 61% patients were male, 71% were heterosexual, and 58% were born in Lima [19].

On the other hand, the imputed HLA alleles for PLHIV and those without HIV in this Peruvian sample were in the range of 196 to 200 alleles (Table 2, Figure 1 and Figure 2). The most frequent alleles for each HLA locus were A*02:01 (35.5%), B*35:01 (11.5%), C*04:01 (22.73%), DPB1*04:02 (29.50%), DQA1*03:01 (22.45%), DQB1*03:419 (28.00%), and DRB1*09:01 (14.50%) in the general population. The Allele Frequency Net Database (http://www.allelefrequencies.net/, accessed on 12 March 2024) [20] reports the frequencies of HLA alleles detected in different populations. In a study carried out in the Uros population localized in Puno City (Peru), the five most frequent alleles were found to be B*35:05 (51.5%), A*02:01 (50%), DQB1*03:02 (38.6%), DQB1*04:02 (33.4%), and DRB1*08:02 (31.9%) [21]. Similar allele frequencies of HLA class I alleles were reported by another study in Lima, with 468 individuals recruited. Of these, 222 were seronegative for HIV-1 (HIV-negative) and 246 were infected with HIV-1. The most common alleles for the different loci were HLA-A*02:01 (46.8%), HLA-B*35:01 (12.0%), and HLA-C*04:01 (37.6%) [22].

We were able to identify significant associations between common HLA variants in our cohort. We identified significant associations between HLA-C*07:01 (*p* = 0.0101, OR = 10.222), HLA-DQA1*03:02 (*p* = 0.0051, OR = 5.297), and HLA-DRB1*09:01 (*p* = 0.0119, OR = 4.788) and susceptibility to HIV infection, while HLA-DQB1*03:419 (*p* = 0.0478, OR = 0.327) was associated with protection from HIV exposure (Table 3). A similar study from Peru concluded that HLA-B*35:43 showed the strongest association with HIV acquisition (*p* = 0.012), while HLA-A*02:01 and HLA-C*04:01 were both associated with high viral loads (*p* = 0.0313 and 0.0001, respectively) [22].

HLA-C belongs to class I and encodes a protein composed of a membrane-bound mature heavy chain and a light chain β2-microglobulin (β2M). HLA-C plays a role in presenting peptides to virus-specific T cells, although much less is known about the CD8+ T cell recognition of peptides restricted by HLA-C. This function is crucial for the initiation and maintenance of adaptive immunity [23]. In addition, our analysis has shown that the HLA-C*07:01 allele might be associated with HIV susceptibility, which is consistent with the results of a genome-wide association study (GWAS) on HIV susceptibility in European individuals [24], where this allelic variant was classified as a risk allele. Another study in a European population showed that the HLA-C*07:01 allele generates susceptibility to autoimmune hepatitis (AIH) [25]. Our study suggests that HLA-C*07:01 is a susceptibility factor in HIV-1 infection.

On the other hand, Killer-cell Immunoglobulin-like Receptor (KIR) genes encode receptors that interact with certain HLA class I molecules to regulate the cytotoxicity of natural killer (NK) cells, playing a key role in the innate immune response against virus-infected cells [26]. Specifically, HLA-C alleles such as HLA-C*07:01 can act as ligands for KIR receptors, modulating immune activity. Certain KIR receptors, like KIR2DL1 and KIR2DS1, recognize HLA-C molecules, which can influence NK cell activation or inhibition and, consequently, the immune response to viral infections [27]. Research shows that specific KIR-HLA interactions improve the control of HIV-1 by enhancing NK cell cytotoxicity and cytokine production [28]. In our findings, HLA-C*07:01, in combination with activating KIR receptors, could potentially modulate the immune response against HIV, potentially explaining its association with increased HIV susceptibility; however, further studies are needed to clarify this relationship.

HLA-DQ molecules form a transmembrane protein composed of an α chain that is encoded by HLA-DQA and consists of α1 and α2 domains, and HLA-DQB encoded a β chain that consists of β1 and β2 domains. Both chains are anchored in the membrane and form an antigen-presenting groove. No association was found between the DQA1*03:02 allele and infectious diseases. However, this allele has been reported to be associated with systemic lupus erythematosus (SLE) in Chinese Han patients [29].

The primary function of HLA class II molecules (HLA-DRB1, HLA-DQA1, and HLA-DQB1) is to process exogenous peptides for presentation to CD4+ T cells, which are crucial in antiviral cellular and humoral immunity [30]. The HLA-DRB1 gene encodes the DRβ1 chain. The association of HLA-DRB1 alleles may affect the specific structure of the HLA-DR molecule and its binding affinity to epitopes [31]. Previous studies have reported associations between the HLA-DRB1*09:01 allele and infectious diseases. For example, Anzurez et al. conducted a study that found that this allelic variant was associated with the risk of severe acute respiratory syndrome coronavirus 2 (SARS-CoV-2) infection in a Japanese population [30]. In contrast, Nguyen et al. reported that HLA-DRB1*09:01 showed a protective effect against the development of Dengue Shock Syndrome (DSS), particularly in patients with DEN-2 infection in a Vietnamese population [32]. Although there is no association between DRB1*09:01 and HIV infection, new associations between the DQA1*03:02 and DRB1*09:01 allelic variants and HIV infection have been identified, which could be attributed to an increased affinity and specificity of the peptide-binding region (PBR), influencing the recognition of pathogen-derived antigens [33].

In contrast to susceptibility alleles, in the HLA-DQB1 locus, the HLA-DQB1*03:419 allele was identified as a novel protective allele against HIV infection in this study. The HLA-DQB1*03:032 and HLA-DQB1*06:02 alleles have shown protective associations against HIV infection in Caucasian and African American ethnic groups, respectively [34]. Rallón et al. showed that the HLA-DQB1*03:02 allele was implicated in protection from HIV infection in a Spanish population, [35] and Hardie et al. showed that the HLA-DQB1*06:03 allele conferred protection from HIV-1 infection in a Pumwani cohort [36]. From a molecular structural perspective, the HLA-DQB1*03:419 allele likely confers resistance by influencing the peptide-binding groove, which determines the specificity and stability of antigen presentation to CD4+ T cells. This allele may affect the binding affinity for particular HIV-derived peptides, potentially enhancing immune recognition and response.

Therefore, the combination of HLA-DQA1 (encodes alpha chain) and HLA-DQB1 (encodes beta chain) forms a binding pocket that determines the DQ molecule’s specificity and diversity for antigen presentation. These allelic variants can affect peptide binding, leading to differential antigen presentation by the DQ molecule, which may be associated with resistance and susceptibility to HIV-1 infection.

In addition to the well-studied role of HLA class I genes in HIV susceptibility, HLA class II genes also play a critical role in modulating immune responses to HIV, particularly through the presentation of viral peptides to CD4+ T cells, which are essential in initiating and sustaining adaptive immune responses. Specific alleles in our study, like HLA-DQA1*03:02 and HLA-DRB1*09:01, may influence HIV susceptibility by enhancing or diminishing the presentation of certain HIV peptides, impacting the CD4+ T cell response and immune coordination [37,38]. For instance, altered peptide binding due to these allelic variants may lead to suboptimal T cell activation or tolerance, which can allow viral persistence and progression. This highlights the potential role of HLA class II alleles, not only in antigen presentation but also in shaping the immune memory and adaptive responses crucial for long-term HIV control.

Our results add information about a population with limited data, namely the Peruvian population, whose genetic composition is represented by that of Lima, the capital city of Peru, with about one third of the country’s population mostly coming from all over the country in the last 70 years. Lima has a mixed population, characterized by a high Amerindian component, of around 70%; the rest are admixed with European, African, and Asian ancestry [39]. This is the first report of the predisposing associations of C*07:01, DQA1*03:02, and DRB1*09:01, and the protective association of DQB1*03:419 with HIV infection in Peruvian patients. One aspect to consider is the lack of significance between the pairs of alleles in the LD analysis, which is intriguing data considering the closeness between the genes, and this may lead us to postulate that other factors and/or interactions, in addition to the independent alleles, would be involved in susceptibility to HIV infection.

The present study has limitations worth noting. First, our no-HIV group was selected based on self-reported risk behavior, which may underestimate actual risk behavior; therefore, we could have introduced biased information. Second, the recruitment methodology did not consider the determination of the patients’ HIV viral load. Thus, we were unable to compare the viral load with other variables. Additionally, the small sample size is also a limitation of this study, although these two parameters did not influence the test results according to the statistical analysis. This study only determined the four-digit resolution prediction for HLA typing. However, a high-throughput HLA typing resolution such as six- or eight-digit resolution is crucial to increasing variant accuracy. We employed high sequencing depth and coverage (≥20X) to generate high-resolution HLA alleles.

These findings highlight the importance of examining HLA allele associations with HIV in underrepresented populations, such as that of Peru, where unique genetic and epidemiological factors may affect HIV susceptibility and progression. Despite global advances in understanding HLA allele variation in HIV outcomes, data specific to Latin American populations remain scarce. Expanding research in diverse Peruvian cohorts will be essential for clarifying these genetic associations, potentially guiding the development of targeted therapeutic strategies and informing HIV management tailored to population-specific immune profiles.

## 5. Conclusions

In conclusion, we identified three HLA alleles (HLA C*07:01, DQA1*03:02, and DRB1*09:01) that were associated with susceptibility to HIV infection, and one HLA allele (HLA DQB1*03:419) that showed a significant protective effect against HIV infection. Our findings contribute to the knowledge of HLA allele diversity in the Peruvian population, with 70% of autochthonous South American ancestry, lay the groundwork for further valuable large-scale use of HLA typing, and offer novel associations with the HIV infection that are relevant to treatment and vaccine studies.

## Figures and Tables

**Table 1:** Sociodemographic, clinical, and ART treatment characteristics of PLHIV and no-HIV groups in Peru.

Characteristics	PLHIV (*n* = 59)	HIV-Uninfected (*n* = 46)	*p*
Sex, *n* (%)			
Female	36 (61.02)	1 (2.17)	0.0000 *^a^
Male	23 (38.98)	45 (97.83)	
Age at entry (Mean ± SD)	41.05 ± 11.08	35.78 ± 10.23	0.0132 *^a^
Initial CD4 cell count (cells/mm^3^), *n* (%)	634.00 ± 291.50	952.67 ± 316.36	0.0000 *^b^
<200	4 (6.78)	0	
200–499	13 (22.03)	2 (4.35)	
≥500	42 (71.19)	44 (95.65)	
Birthplace, *n* (%)			
Central Coast region: Lima	37 (62.71)	38 (82.61)	
North Coast region	15 (25.42)	5 (10.87)	
Andean region	7 (11.86)	1 (2.17)	
Amazon region	0	2 (4.35)	
Use of ART, *n* (%)			
Yes	59 (100.00) **	-	
No	-	-	
ART regimen, *n* (%)			
2 PIs + 1 IIs	1 (1.69)	-	
2 PIs + 2 NRTIs	5 (8.47)	-	
2 NRTIs + 1 IIs	24 (40.68)	-	
2 NRTIs + 1 NNRTIs	26 (44.07)	-	
Drugs, *n* (%)			
NRTIs			
Lamivudine (3TC)	50 (84.75)	-	
Tenofovir (TDF)	51 (86.44)	-	
Emtricitabine (FTC)	5 (8.47)	-	
Abacavir (ABC)	3 (5.08)	-	
Zidovudine (AZT)	1 (1.69)	-	
NNRTIs			
Efavirenz (EFV)	26 (44.07)	-	
PIs			
Ritonavir (RTV)	6 (10.17)	-	
Lopinavir (LPV)	4 (6.78)	-	
Atazanavir (ATV)	1 (1.69)	-	
Darunavir (DRV)	1 (1.69)	-	
IIs			
Dolutegravir (DTG)	21 (35.59)	-	
Raltegravir (RAL)	4 (6.78)	-	
Sexual Orientation, *n* (%)			
Homosexual	-	14 (30.43)	
Heterosexual	-	26 (56.52)	
Bisexual	-	6 (13.04)	
Sex encounters with sex workers, *n* (%)			
Yes	-	46 (100.00)	
No	-	-	
No. of sexual partners in the last 12 months			
Less than 5 sexual partners	-	3 (6.52)	
5–15 sexual partners	-	35 (76.09)	
More than 15 sexual partners	-	8 (17.39)	

^a^ Pearson’s chi-squared test; ^b^ Mann–Whitney U test; * *p*-value < 0.05. ART: antiretroviral therapy; PLHIV: people living with HIV; NNRTIs: non-nucleoside reverse transcriptase inhibitors; NRTIs: nucleoside reverse transcriptase inhibitors; PIs: protease inhibitors; IIs: integrase inhibitors. ** Two patients did not specify their medications.

**Table 2:** Summary of imputed HLA loci for PLHIV and individuals without HIV in Peru.

HLA Locus	Number of Alleles		Alleles by Locus, Total
	PLHIV (*n* = 56)	Without HIV (*n* = 44)	
HLA-A	112	88	200
HLA-B	112	88	200
HLA-C	110	88	198
HLA-DPB1	112	88	200
HLA-DQA1	108	88	196
HLA-DQB1	112	88	200
HLA-DRB1	112	88	200

**Table 3:** Haplotype frequencies of HLA class I and II systems and the association of PLHIV and those without HIV in Peru.

HLA Alleles	Allele Frequency		Allele Effect		
	PLHIV	Without HIV	OR (95% CI)	*p*	*p* Perm
**Susceptible**					
C*07:01	0.1182	0.0227	10.222 (1.40–74.55)	0.0219	0.0101
DQA1*03:02	0.1481	0.0568	5.2972 (1.48–19.02)	0.0106	0.0051
DRB1*09:01	0.1696	0.1136	4.7880 (1.39–16.44)	0.0129	0.0119
**Protective**					
DQB1*03:419	0.2232	0.3523	0.3273 (0.11–0.96)	0.0412	0.0478

OR: odds ratio; CI: confidence interval. *p* values were calculated using logistic regression with age, sex, CD4 covariates. The corrected *p* values (*p* perm) were calculated using 10,000 permutations. A *p*-value < 0.05 was considered statistically significant.

## Data Availability

The data that support the findings of this study are available in Zenodo.org at 10.5281/zenodo.14010955.

## Supplementary Materials

The following supporting information can be downloaded at https://www.mdpi.com/article/10.3390/v16111708/s1, Table S1: Complete allele frequencies detected in study population for each locus.; Table S2: Allele frequency distribution of HLA class I and II systems and his association between PLHIV (cases) and HIV-uninfected (controls) in Peru.

## References

[1] Unaids (2023). UNAIDS 2023 Data. https://www.unaids.org/sites/default/files/media_asset/UNAIDS_FactSheet_en.pdf.

[2] Horton R., Wilming L., Rand V., Lovering R.C., Bruford E.A., Khodiyar V.K., Lush M.J., Povey S., Talbot C.C., Wright M.W. (2004). Gene map of the extended human MH. C. Nat. Rev. Genet..

[3] Kim J.Y., Lee S.Y., Kim G.G., Song H.I., Jang M.M., Lee C.S., Hong J.Y., Shin M.G., Choi H.J. (2023). Validation and application of new NGS-based HLA genotyping to clinical diagnostic practice. HLA.

[4] Robinson J., Barker D.J., Georgiou X., Cooper M.A., Flicek P., Marsh S.G.E. (2020). IPD-IMGT/HLA Database. Nucleic Acids Res..

[5] Marsh S.G., Albert E.D., Bodmer W.F., Bontrop R.E., Dupont B., Erlich H.A., Fernández-Viña M., Geraghty D.E., Holdsworth R., Hurley C.K. (2010). Nomenclature for factors of the HLA system, 2010. Tissue Antigens.

[6] Luo M. (2022). Natural Immunity against HIV-1: Progression of Understanding after Association Studies. Viruses.

[7] Dashti M., Malik M.Z., Nizam R., Jacob S., Al-Mulla F., Thanaraj T.A. (2024). Evaluation of HLA typing content of next-generation sequencing datasets from family trios and individuals of arab ethnicity. Front. Genet..

[8] Bentley G., Higuchi R., Hoglund B., Goodridge D., Sayer D., Trachtenberg E.A., Erlich H.A. (2009). High-resolution, high-throughput HLA genotyping by next-generation sequencing. Tissue Antigens.

[9] Erlich H. (2012). HLA DNA typing: Past, present, and future. Tissue Antigens.

[10] Miller S., Dykes D., Polesky H. (1988). A simple salting out procedure for extracting DNA from human nucleated cells. Nucleic Acids Res..

[11] Chen S., Zhou Y., Chen Y., Gu J. (2018). Fastp: An ultra-fast all-in-one FASTQ preprocessor. Bioinformatics.

[12] Li H., Durbin R. (2009). Fast and accurate short read alignment with Burrows-Wheeler transform. Bioinformatics.

[13] Li H., Handsaker B., Wysoker A., Fennell T., Ruan J., Homer N., Marth G., Abecasis G., Durbin R. (2009). The Sequence Alignment/Map format and SAMtools. Bioinformatics.

[14] Szolek A., Schubert B., Mohr C., Sturm M., Feldhahn M., Kohlbacher O. (2014). OptiType: Precision HLA typing from next-generation sequencing data. Bioinformatics.

[15] Orenbuch R., Filip I., Comito D., Shaman J., Pe’er I., Rabadan R. (2020). arcasHLA: High-resolution HLA typing from RNAseq. Bioinformatics.

[16] Fan Y., Song Y.Q. (2017). PyHLA: Tests for the association between HLA alleles and diseases. BMC Bioinform..

[17] Liu P., Yao M., Gong Y., Song Y., Chen Y., Ye Y., Liu X., Li F., Dong H., Meng R. (2021). Benchmarking the Human Leukocyte Antigen Typing Performance of Three Assays and Seven Next-Generation Sequencing-Based Algorithms. Front. Immunol..

[18] Sanchez-Mazas A. (2020). A review of HLA allele and SNP associations with highly prevalent infectious diseases in human populations. Swiss Med. Wkly..

[19] Amanzo-Vargas M.P., Arellano-Veintemilla T., González-Lagos E., Echevarría J., Mejía F., Graña A., Gotuzzo E. (2023). Socio-Demographic, Clinical, and Mortality Differences between HIV-Infected and HIV/HTLV-1 Co-Infected Patients in Peru. Pathogens.

[20] Gonzalez-Galarza F.F., McCabe A., Santos E.J., Jones J., Takeshita L.Y., Ortega-Rivera N.D., Del Cid-Pavon G.M., Ramsbottom K., Ghattaoraya G.S., Alfirevic A. (2020). Allele frequency net database (AFND) 2020 update: Gold-standard data classification, open access genotype data and new query tools. Nucleic Acid. Res..

[21] Arnaiz-Villena A., Gonzalez-Alcos V., Serrano-Vela J.I., Reguera R., Barbolla L., Parga-Lozano C., Gómez-Prieto P., Abd-El-Fatah-Khalil S., Moscoso J. (2009). HLA genes in Uros from Titikaka Lake, Peru: Origin and relationship with other Amerindians and worldwide populations. Int. J. Immunogenet..

[22] Olvera A., Pérez-Álvarez S., Ibarrondo J., Ganoza C., Lama J.R., Lucchetti A., Cate S., Hildebrand W., Bernard N., Gomez L. (2015). The HLA-C*04: 01/KIR2DS4 gene combination and human leukocyte antigen alleles with high population frequency drive rate of HIV disease progression. AIDS.

[23] Blais M.E., Dong T., Rowland-Jones S. (2011). HLA-C as a mediator of natural killer and T-cell activation: Spectator or key player?. Immunology.

[24] Pereyra F., Jia X., McLaren P.J., Telenti A., de Bakker P.I., Walker B.D., Ripke S., Brumme C.J., Pulit S.L., Carrington M. (2010). The major genetic determinants of HIV-1 control affect HLA class I peptide presentation. Science.

[25] Strettell M.D., Thomson L.J., Donaldson P.T., Bunce M., O’Neill C.M., Williams R. (1997). HLA-C genes and susceptibility to type 1 autoimmune hepatitis. Hepatology.

[26] Qi Y., Martin M.P., Gao X., Jacobson L., Goedert J.J., Buchbinder S., Kirk G.D., O’Brien S.J., Trowsdale J., Carrington M. (2006). KIR/HLA pleiotropism: Protection against both HIV and opportunistic infections. PLoS Pathog..

[27] Vargas L.B., Dourado R.M., Amorim L.M., Ho B., Calonga-Solís V., Issler H.C., Marin W.M., Beltrame M.H., Petzl-Erler M.L., Hollenbach J.A. (2020). Single Nucleotide Polymorphism in KIR2DL1 Is Associated With HLA-C Expression in Global Populations. Front. Immunol..

[28] Boudreau J.E., Hsu K.C. (2018). Natural killer cell education in human health and disease. Curr. Opin. Immunol..

[29] Qian J., Chen Y., Yang X., Wang Q., Zhao J., Deng X., Ding Y., Li S., Liu Y., Tian Z. (2023). Association Study Identified HLA-DQA1 as a Novel Genetic Risk of Systemic Lupus Erythematosus-Associated Pulmonary Arterial Hypertension. Arthritis Rheumatol..

[30] Anzurez A., Naka I., Miki S., Nakayama-Hosoya K., Isshiki M., Watanabe Y., Nakamura-Hoshi M., Seki S., Matsumura T., Takano T. (2021). Association of HLA-DRB1*09:01 with severe COVID-19. HLA.

[31] Choo S.Y. (2007). The HLA system: Genetics, immunology, clinical testing, and clinical implications. Yonsei Med. J..

[32] Nguyen T.P.L., Kikuchi M., Huong V.T.Q., Ha D.H., Thuy T.T., Tham V.D., Tuan H.M., Tuong V.V., Nga C.T.P., Dat T.V. (2008). Protective and enhancing HLA alleles, HLA-DRB1*0901 and HLA-A*24, for severe forms of dengue virus infection, dengue hemorrhagic fever and dengue shock syndrome. PLoS Negl. Trop. Dis..

[33] Sarri C.A., Giannoulis T., Moutou K.A., Mamuris Z. (2021). HLA class II peptide-binding-region analysis reveals funneling of polymorphism in action. Immunol. Lett..

[34] Roe D.L., Lewis R.E., Cruse J.M. (2000). Association of HLA-DQ and -DR alleles with protection from or infection with HIV-1. Exp. Mol. Pathol..

[35] Rallón N., Restrepo C., Vicario J., Romero J., Rodríguez C., García-Samaniego J., García M., Cabello A., Gorgolas M., Benito L.M. (2017). Human leucocyte antigen (HLA)-DQB1*03:02 and HLA-A*02:01 have opposite patterns in their effects on susceptibility to HIV infection. HIV Med..

[36] Hardie R., Luo M., Bruneau B., Knight E., Nagelkerke N., Kimani J., Wachihi C., Ngugi E., Plummer F. (2008). Human leukocyte antigen-DQ alleles and haplotypes and their associations with resistance and susceptibility to HIV-1 infection. AIDS.

[37] Qian L., Zhang H., Meng Y., Ji Q., Zhou L., Zhang L. (2019). The association between HLA-DRB1 and systemic lupus erythematosus: A systematic review and meta-analysis. Sci. Rep..

[38] Anzurez A., Bello-López J.M., Rodríguez-Serrano E., Gómez-Martin D., De la Chesnaye E. (2021). HLA associations and clinical outcomes in SARS-CoV-2 infection. Human. Immunol..

[39] Sandoval J., Salazar-Granara A., Acosta O., Castillo-Herrera W., Fujita R., Pena S.D., Santos F.R. (2013). Tracing the genomic ancestry of Peruvians reveals a major legacy of pre-Columbian ancestors. J. Hum. Genet..

