# Supplementary material for: New Associations with the HIV Predisposing and Protective Alleles of the Human Leukocyte Antigen System in a Peruvian Population
Source: Viruses. 2024 Oct 30;16(11):1708. doi: 10.3390/v16111708 (PMC11598887; doi:10.3390/v16111708)
Supplement: Supplementary file 1 [file viruses-16-01708-s001.zip › viruses-3255850-supplementary.pdf]

## New Associations with HIV Predisposing and Protective Alleles of the HLA System in a Peruvian Population

**Supplementary Table S1. Complete allele frequencies detected in study population for each locus.**

| HLA-A | Freq | HLA-B | Freq | HLA-C | Freq | HLA-DPB1 | Freq | HLA-DQA1 | Freq | HLA-DQB1 | Freq | HLA-DRB1 | Freq |
|---|---|---|---|---|---|---|---|---|---|---|---|---|---|
| A*01:01 | 0.020 | B*07:02 | 0.050 | C*01:02 | 0.121 | DPB1*02:01 | 0.070 | DQA1*01:01 | 0.051 | DQB1*02:01 | 0.060 | DRB1*01:01 | 0.010 |
| A*01:02 | 0.005 | B*08:01 | 0.020 | C*01:17 | 0.005 | DPB1*02:02 | 0.005 | DQA1*01:02 | 0.082 | DQB1*02:02 | 0.005 | DRB1*01:02 | 0.020 |
| A*02:01 | 0.355 | B*13:02 | 0.005 | C*02:02 | 0.030 | DPB1*03:01 | 0.040 | DQA1*01:03 | 0.010 | DQB1*02:188 | 0.005 | DRB1*01:03 | 0.010 |
| A*02:02 | 0.005 | B*13:03 | 0.005 | C*03:02 | 0.015 | DPB1*04:01 | 0.160 | DQA1*01:04 | 0.010 | DQB1*02:197 | 0.010 | DRB1*03:01 | 0.030 |
| A*02:03 | 0.010 | B*14:01 | 0.005 | C*03:03 | 0.025 | DPB1*04:02 | 0.295 | DQA1*01:05 | 0.026 | DQB1*02:59 | 0.005 | DRB1*04:01 | 0.005 |
| A*02:04 | 0.005 | B*14:02 | 0.030 | C*03:04 | 0.071 | DPB1*05:01 | 0.040 | DQA1*01:06 | 0.005 | DQB1*03:02 | 0.210 | DRB1*04:02 | 0.010 |
| A*02:05 | 0.005 | B*15:01 | 0.020 | C*04:01 | 0.227 | DPB1*09:01 | 0.005 | DQA1*01:48 | 0.005 | DQB1*03:03 | 0.050 | DRB1*04:03 | 0.025 |
| A*02:06 | 0.005 | B*15:02 | 0.005 | C*04:03 | 0.005 | DPB1*104:01 | 0.015 | DQA1*02:01 | 0.046 | DQB1*03:22 | 0.015 | DRB1*04:04 | 0.065 |
| A*02:10 | 0.005 | B*15:04 | 0.060 | C*05:01 | 0.020 | DPB1*105:01 | 0.005 | DQA1*02:17 | 0.010 | DQB1*03:229 | 0.005 | DRB1*04:07 | 0.130 |
| A*02:11 | 0.100 | B*15:10 | 0.005 | C*06:02 | 0.015 | DPB1*107:01 | 0.045 | DQA1*03:01 | 0.225 | DQB1*03:251 | 0.015 | DRB1*04:10 | 0.020 |
| A*02:13 | 0.020 | B*15:17 | 0.005 | C*07:01 | 0.076 | DPB1*1082:01 | 0.005 | DQA1*03:02 | 0.107 | DQB1*03:419 | 0.280 | DRB1*04:11 | 0.015 |
| A*02:22 | 0.045 | B*15:39 | 0.010 | C*07:02 | 0.187 | DPB1*1083:01 | 0.005 | DQA1*03:03 | 0.077 | DQB1*03:49 | 0.005 | DRB1*07:01 | 0.030 |
| A*02:33 | 0.005 | B*15:42 | 0.010 | C*07:05 | 0.005 | DPB1*10:01 | 0.010 | DQA1*03:15 | 0.005 | DQB1*03:95N | 0.010 | DRB1*08:02 | 0.110 |
| A*02:58 | 0.005 | B*18:01 | 0.015 | C*07:18 | 0.005 | DPB1*11:01 | 0.020 | DQA1*04:01 | 0.112 | DQB1*03:96 | 0.100 | DRB1*08:03 | 0.010 |
| A*02:60 | 0.005 | B*27:05 | 0.005 | C*08:01 | 0.040 | DPB1*1243:01 | 0.005 | DQA1*05:01 | 0.026 | DQB1*03:99Q | 0.015 | DRB1*08:04 | 0.015 |
| A*03:01 | 0.045 | B*35:01 | 0.115 | C*08:02 | 0.015 | DPB1*126:01 | 0.010 | DQA1*05:03 | 0.117 | DQB1*04:02 | 0.040 | DRB1*08:18 | 0.015 |
| A*03:05 | 0.005 | B*35:02 | 0.020 | C*08:03 | 0.046 | DPB1*1317:01Q | 0.005 | DQA1*05:05 | 0.061 | DQB1*04:52 | 0.005 | DRB1*09:01 | 0.145 |
| A*11:01 | 0.020 | B*35:04 | 0.015 | C*08:27 | 0.005 | DPB1*133:01 | 0.010 | DQA1*05:48 | 0.020 | DQB1*05:01 | 0.045 | DRB1*09:21 | 0.025 |
| A*23:01 | 0.005 | B*35:05 | 0.040 | C*12:02 | 0.010 | DPB1*135:01 | 0.005 | DQA1*06:01 | 0.005 | DQB1*05:03 | 0.020 | DRB1*10:01 | 0.015 |
| A*24:02 | 0.110 | B*35:09 | 0.010 | C*12:03 | 0.020 | DPB1*13:01 | 0.010 | | | DQB1*05:226 | 0.005 | DRB1*11:01 | 0.015 |
| A*24:03 | 0.010 | B*35:14 | 0.010 | C*14:02 | 0.005 | DPB1*14:01 | 0.135 | | | DQB1*05:296 | 0.005 | DRB1*11:02 | 0.010 |
| A*25:01 | 0.015 | B*35:20 | 0.005 | C*15:02 | 0.030 | DPB1*17:01 | 0.005 | | | DQB1*06:01 | 0.005 | DRB1*11:04 | 0.020 |
| A*26:01 | 0.015 | B*35:22 | 0.005 | C*15:05 | 0.005 | DPB1*184:01 | 0.005 | | | DQB1*06:02 | 0.040 | DRB1*12:01 | 0.005 |
| A*30:01 | 0.010 | B*35:25 | 0.005 | C*16:02 | 0.005 | DPB1*18:01 | 0.005 | | | DQB1*06:03 | 0.005 | DRB1*12:02 | 0.005 |
| A*30:02 | 0.015 | B*35:31 | 0.010 | C*17:01 | 0.010 | DPB1*21:01 | 0.005 | | | DQB1*06:04 | 0.015 | DRB1*13:02 | 0.030 |
| A*30:04 | 0.005 | B*35:43 | 0.010 | | | DPB1*23:01 | 0.005 | | | DQB1*06:09 | 0.010 | DRB1*13:03 | 0.010 |
| A*31:01 | 0.035 | B*38:01 | 0.050 | | | DPB1*352:01 | 0.005 | | | DQB1*06:112N | 0.005 | DRB1*14:02 | 0.090 |
| A*31:02 | 0.005 | B*39:01 | 0.040 | | | DPB1*40:01 | 0.005 | | | DQB1*06:118 | 0.005 | DRB1*14:06 | 0.005 |
| A*31:04 | 0.005 | B*39:05 | 0.020 | | | DPB1*45:01 | 0.005 | | | DQB1*06:44 | 0.005 | DRB1*14:54 | 0.020 |

| | | | | | | | | | | | | | |
|---|---|---|---|---|---|---|---|---|---|---|---|---|---|
| A*32:01 | 0.010 | B*39:06 | 0.025 | | | DPB1*585:01 | 0.005 | | | | | DRB1*15:01 | 0.025 |
| A*33:01 | 0.020 | B*39:09 | 0.030 | | | DPB1*651:01 | 0.035 | | | | | DRB1*15:03 | 0.015 |
| A*33:03 | 0.005 | B*39:13 | 0.020 | | | DPB1*835:01 | 0.005 | | | | | DRB1*16:01 | 0.005 |
| A*34:02 | 0.005 | B*40:01 | 0.005 | | | DPB1*882:01 | 0.005 | | | | | DRB1*16:02 | 0.040 |
| A*68:01 | 0.040 | B*40:02 | 0.040 | | | DPB1*954:01 | 0.005 | | | | | | |
| A*68:02 | 0.010 | B*40:04 | 0.045 | | | DPB1*968:01 | 0.005 | | | | | | |
| A*69:01 | 0.005 | B*40:08 | 0.005 | | | | | | | | | | |
| A*80:01 | 0.010 | B*40:09 | 0.005 | | | | | | | | | | |
| | | B*41:01 | 0.005 | | | | | | | | | | |
| | | B*44:02 | 0.010 | | | | | | | | | | |
| | | B*44:03 | 0.010 | | | | | | | | | | |
| | | B*46:01 | 0.005 | | | | | | | | | | |
| | | B*48:01 | 0.065 | | | | | | | | | | |
| | | B*48:04 | 0.005 | | | | | | | | | | |
| | | B*49:01 | 0.020 | | | | | | | | | | |
| | | B*51:01 | 0.040 | | | | | | | | | | |
| | | B*51:08 | 0.005 | | | | | | | | | | |
| | | B*51:13 | 0.005 | | | | | | | | | | |
| | | B*52:01 | 0.010 | | | | | | | | | | |
| | | B*53:01 | 0.020 | | | | | | | | | | |
| | | B*54:01 | 0.005 | | | | | | | | | | |
| | | B*55:01 | 0.005 | | | | | | | | | | |
| | | B*57:03 | 0.005 | | | | | | | | | | |

**Supplementary Table S2. Allele frequency distribution of HLA class I and II systems and his association between PLHIV (cases) and HIV-uninfected (controls) in Peru.**

| HLA alleles | Allele frequency | | Allele effect | | | |
|---|---|---|---|---|---|---|
| | PLHIV | HIV-uninfected | OR | CI | *p* | *p* adj |
| A*02:01 | 0.339 | 0.375 | 0.866 | 0.4302-1.7441 | 0.687 | 0.687 |
| A*02:11 | 0.134 | 0.057 | 2.273 | 0.6852-7.5384 | 0.180 | 0.453 |
| A*24:02 | 0.107 | 0.114 | 1.768 | 0.5995-5.2154 | 0.302 | 0.453 |
| B*15:04 | 0.054 | 0.068 | 0.880 | 0.1826-4.2383 | 0.873 | 0.873 |
| B*35:01 | 0.116 | 0.114 | 0.884 | 0.2826-2.7639 | 0.832 | 0.873 |
| B*48:01 | 0.054 | 0.080 | 0.723 | 0.1929-2.7057 | 0.630 | 0.873 |
| C*01:02 | 0.091 | 0.159 | 1.015 | 0.3582-2.8781 | 0.977 | 0.977 |
| C*03:04 | 0.082 | 0.057 | 0.549 | 0.1301-2.3179 | 0.415 | 0.691 |
| C*04:01 | 0.236 | 0.216 | 1.192 | 0.52-2.7332 | 0.678 | 0.848 |
| C*07:01 | 0.118 | 0.023 | 10.222 | 1.4014-74.5521 | **0.022** | 0.109 |
| C*07:02 | 0.182 | 0.193 | 1.555 | 0.5866-4.1221 | 0.375 | 0.691 |
| DPB1*02:01 | 0.080 | 0.057 | 0.556 | 0.0844-3.6697 | 0.542 | 0.542 |
| DPB1*04:01 | 0.188 | 0.125 | 1.835 | 0.689-4.8881 | 0.225 | 0.449 |
| DPB1*04:02 | 0.277 | 0.318 | 1.395 | 0.6514-2.9863 | 0.392 | 0.522 |
| DPB1*14:01 | 0.098 | 0.182 | 0.517 | 0.1862-1.4331 | 0.205 | 0.449 |
| DQA1*01:01 | 0.056 | 0.046 | 0.843 | 0.1452-4.8932 | 0.849 | 0.849 |
| DQA1*01:02 | 0.120 | 0.034 | 1.959 | 0.4673-8.2107 | 0.358 | 0.568 |
| DQA1*03:01 | 0.232 | 0.216 | 1.170 | 0.5031-2.7201 | 0.716 | 0.818 |
| DQA1*03:02 | 0.148 | 0.057 | 5.297 | 1.4752-19.0213 | **0.011** | 0.085 |
| DQA1*03:03 | 0.056 | 0.102 | 0.351 | 0.074-1.668 | 0.188 | 0.376 |
| DQA1*04:01 | 0.074 | 0.159 | 0.352 | 0.0893-1.3855 | 0.135 | 0.376 |
| DQA1*05:03 | 0.093 | 0.148 | 0.629 | 0.2015-1.9664 | 0.426 | 0.568 |
| DQA1*05:05 | 0.037 | 0.091 | 0.317 | 0.0577-1.7399 | 0.186 | 0.376 |
| DQB1*02:01 | 0.080 | 0.034 | 2.700 | 0.4822-15.1198 | 0.258 | 0.258 |
| DQB1*03:02 | 0.241 | 0.171 | 2.128 | 0.77-5.8808 | 0.145 | 0.258 |
| DQB1*03:419 | 0.223 | 0.352 | 0.327 | 0.112-0.9563 | **0.041** | 0.165 |
| DQB1*03:96 | 0.071 | 0.136 | 0.454 | 0.1243-1.6603 | 0.233 | 0.258 |
| DRB1*04:04 | 0.063 | 0.068 | 3.059 | 0.5937-15.7629 | 0.181 | 0.302 |
| DRB1*04:07 | 0.125 | 0.136 | 1.015 | 0.3323-3.0992 | 0.979 | 0.979 |
| DRB1*08:02 | 0.071 | 0.159 | 0.347 | 0.087-1.384 | 0.134 | 0.302 |
| DRB1*09:01 | 0.170 | 0.114 | 4.788 | 1.3941-16.4434 | **0.013** | 0.064 |
| DRB1*14:02 | 0.063 | 0.125 | 0.625 | 0.1837-2.1229 | 0.451 | 0.563 |

OR: odds ratio; CI: confidence interval. P-values were calculated using a logistic regression model. The adjusted p-values (P adj) were calculated using 10,000 permutation results and by imposing a false discovery rate (FDR) of 0.05. A p-value < 0.05 was considered statistically significant (highlighted in bold).
